# Supplementary material for: Comprehensive Annotation and Functional Exploration of MicroRNAs in Lettuce
Source: Front Plant Sci. 2021 Dec 24;12:781836. doi: 10.3389/fpls.2021.781836 (PMC8739914; doi:10.3389/fpls.2021.781836)
Supplement: Supplementary file 3 [file Data_Sheet_9.ZIP › GSM2230752.plot/Lsa-miRN1664_Lsat_1_v5_gn_7_48240.1_659_TPlot.pdf]

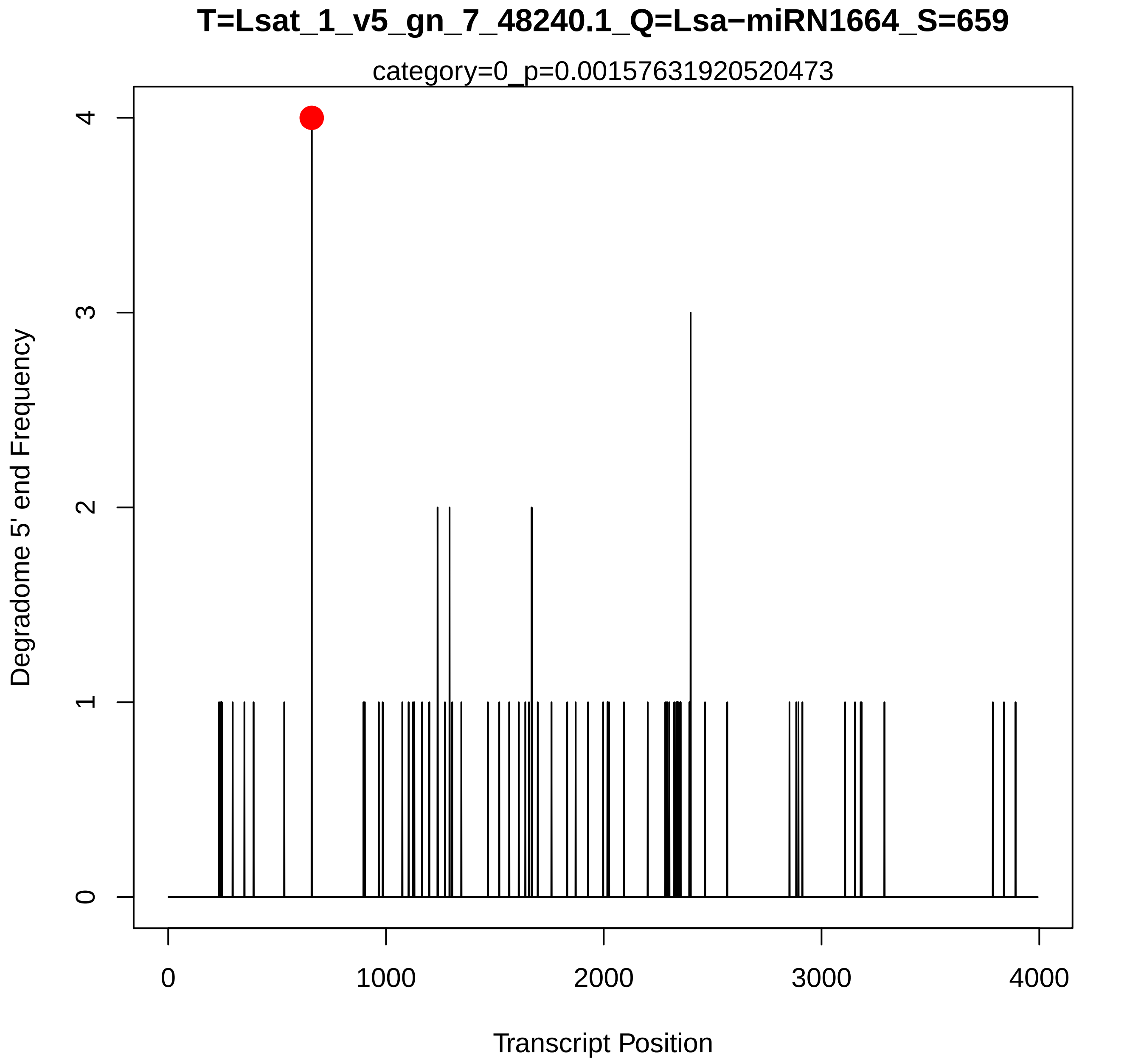

T=Lsat_1_v5_gn_7_48240.1_Q=Lsa−miRN1664_S=659
category=0_p=0.00157631920520473
Degradome 5' end Frequency
Transcript Position
0
1
2
3
4
0
1000
2000
3000
4000
